# The clinical evidence for postbiotics as microbial therapeutics

**DOI:** 10.1080/19490976.2022.2117508

**Published:** 2022-10-02

**Authors:** Alexis Mosca, Ana Teresa Abreu Y Abreu, Kok Ann Gwee, Gianluca Ianiro, Jan Tack, Thi Viet Ha Nguyen, Colin Hill

**Affiliations:** a Pediatric Gastroenterology and Nutrition Department, APHP Robert Debré, Paris, France; b Gastroenterologist and Neuro-gastroenterologist, Angeles del Pedregal Hospital, Mexico City, Mexico; c Department of Medicine, Yong Loo Lin School of Medicine, National University of Singapore and Gleneagles Hospital, Singapore; d Gastroenterology Unit, Fondazione Policlinico Universitario”A. Gemelli” IRCCS, Rome, Italy; e Department of Gastroenterology, University Hospitals Leuven, Belgium; f Department of Gastroenterology, National Children’s Hospital, Vietnam; g APC Microbiome Institute, University College Cork, Ireland

**Keywords:** Microbiota, postbiotics, microbial therapeutics, clinical benefits, gastrointestinal disorders, allergy, upper respiratory tract infection, stress, metabolic syndrome

## Abstract

An optimally operating microbiome supports protective, metabolic, and immune functions, but disruptions produce metabolites and toxins which can be involved in many conditions. Probiotics have the potential to manage these. However, their use in vulnerable people is linked to possible safety concerns and maintaining their viability is difficult. Interest in postbiotics is therefore increasing. Postbiotics contain inactivated microbial cells or cell components, thus are more stable and exert similar health benefits to probiotics. To review the evidence for the clinical benefits of postbiotics in highly prevalent conditions and consider future potential areas of benefit. There is growing evidence revealing the diverse clinical benefits of postbiotics in many prevalent conditions. Postbiotics could offer a novel therapeutic approach and may be a safer alternative to probiotics. Establishing interaction mechanisms between postbiotics and commensal microorganisms will improve the understanding of potential clinical benefits and may lead to targeted postbiotic therapy.

## Introduction

The human microbiome is the catalog of all microorganisms inhabiting the human body and their genetic complement.^1^ When operating optimally, the microbiome plays an important role in human health by supporting protective, metabolic, and immune functions.^2^ Specifically, the evidence suggests that the relationship between the gut microbiome and intestinal epithelial cells supports mucosal and systemic immunity, neuroendocrine function, and intestinal and extra-intestinal health from infancy to adulthood.^3–5^ When the microbiome is disrupted, metabolites and toxins are produced and are involved with both intestinal and extraintestinal diseases, including chronic digestive disorders, chronic inflammatory disorders, autoimmunity, allergies, and metabolic syndromes.^6,7^ Microbiome disruption can also influence disease development in distal organs including the brain, liver, lung, and adipose tissue.^7^

In recent years, it has been suggested that live microorganisms with bioactive properties (probiotics or live biotherapeutics) have therapeutic potential for various immune, neurological, and physiological pathologies.^2,8^ However, there have been some concerns about administering live microbial therapeutics to immunocompromised or critically ill individuals, those with intestinal barrier dysfunction, or neonates and young children.^9^ These concerns include the risk of translocation from the gut into the blood, the risk of acquiring and transferring antibiotic resistance genes and the risk of interfering with normal colonization of neonatal gut microbiota.^9,10^ Furthermore, live biotherapeutic viability is difficult to maintain and can be unstable at room temperature, shortening shelf-life. These issues have therefore increased the interest in using alternative biotherapeutic products containing inanimate bacteria, microorganism-derived cell components and metabolites which are safer to use in vulnerable populations. These biotherapeutic products are referred to as postbiotics.

Postbiotics are defined as “a preparation of inanimate microorganisms and/or their components that confers a health benefit on the host”^11^ and are produced from inactivated commensal bacteria. They include inactivated microbial cells, cell-free supernatants, and key components, commonly inactivated by heat. Although inanimate, they exert similar, and sometimes more, health benefits compared with probiotics, a phenomenon that has been referred to as the “probiotic paradox”.^12^

Their efficacy is thought to be derived from interactions between the host and products generated by the microbiome during microbial growth following alterations caused by the postbiotic. These products include microbial metabolites, proteins, lipids, carbohydrates, vitamins, organic acids, cell wall components or other complex molecules.^13,14^ It is also possible that active molecules in the postbiotic preparation may pass through the mucus layers and stimulate epithelial cells more directly.^9^ Furthermore, the loss of viability and cell lysis could potentially produce more complex beneficial effects such as immunomodulation.^9^

Being inanimate, their efficacy is not dependent on cell viability meaning they can be used in combination with antimicrobials without losing efficacy.^10,15^ In addition, their inanimate nature means they are less sensitive to environmental conditions resulting in a longer shelf-life and enabling storage and transportation at ambient temperatures.^3,10,11,16,17^ Postbiotics have the potential to provide novel therapeutic approaches and may pave the way towards increasing the potency of active microorganisms or provide a means to convert them into functional ingredients.^18^

This paper aims to review the available evidence for the clinical benefits of postbiotics in highly prevalent conditions and consider further potential areas of benefit.

## Overview of knowledge about mechanism of action

**Figure 1.**
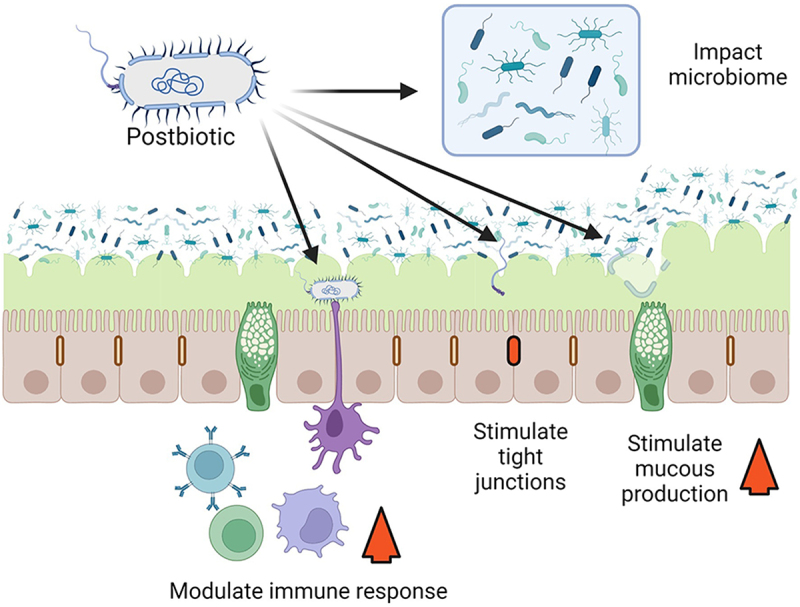
**Postbiotic mechanism of action**. Postbiotics could act in many ways, four of which are illustrated here. Postbiotics could enhance barrier function, through the stimulation of tight junctions, or by stimulating mucous production. Postbiotics could also act through changes in the microbiome and could modulate the immune response. Created with BioRender.

Postbiotics are a complex preparation containing many bioactive compounds with multiple mechanisms of action. The mechanisms of action by which postbiotics exert their benefit and their role in human health are not clearly understood in most instances. These mechanisms may occur independently or in combination and in some cases could be similar to known probiotic mechanisms of action.^11,19,20^ Two of the major mechanisms by which postbiotics could potentiate a clinical benefit are immune system modulation and enhancing intestinal barrier function (Figure 1). We will only provide a brief overview of mechanisms of action because they are covered in detail in other publications. ^11^

Local and systemic immunomodulation generally occurs by stimulating various cytokine and immune modulator expression and microorganism-associated molecular patterns interacting with specific immune cell receptors such as C-type lectins and toll-like receptors (TLR).^21^ Other immunomodulatory microbial metabolites including histamine, branched chain fatty acids and short-chain fatty acids (SCFA), and cell wall components including peptidoglycans and muramic acid may be contained in postbiotics and can influence various immune responses.^22,23^

Intestinal barrier function can be enhanced by exopolysaccharides, including those from *Bifidobacterium* spp.^24^ Some *Bifidobacterium* spp. have also been shown to promote tight junction function.^25^ Furthermore, if present in sufficient amounts, SCFAs can potentially modify barrier function and protect against lipopolysaccharide-induced disruption.^26^ Additionally, postbiotics may modulate the intestinal microbiome itself. For example, lactic acid and bacteriocins can have a direct antimicrobial effect,^27,28^ while indirect microbiome modulation can occur through mechanisms such as carrying lactic acid that is consumed by microbiome microorganisms resulting in SCFAs and butyrate production which both have beneficial roles.^29^

Postbiotic preparations could also antagonize intestinal pathogens by delivering antimicrobial compounds (metabolites and bacteriocins) that may prevent biofilm formation and inactivate specific microorganisms.^11,30^

**Table 1.** Clinical evidence for postbiotics as microbial therapeutics through intestinal barrier function enhancement.

Study design	Condition	Population	Postbiotic	Results
**Evidence of efficacy in gastrointestinal disorders**				
Randomized, placebo-controlled trial ^39^	Acute watery rotavirus diarrhea	73 children aged 3 to 24 months.	Inactivated *Lactobacillus* LB plus fermented culture medium	Significantly shorter duration of diarrhea compared with placebo. Fewer rotavirus positive children had watery stools after 24 hours in the *Lactobacillus* group.
Randomized, double-blind, placebo-controlled trial^40^	Non-rotavirus diarrhea	Hospitalized infants aged 1–24 months	Inactivated *Lactobacillus* LB plus fermented culture medium	*Lactobacillus* LB significantly shortened diarrhea duration by 1 d (treatment group = 39.5 h ± 10.5, placebo group = 63.4 h ± 14.9, p = <0.01).
Randomized, double-blind, controlled trial^41^	Acute watery diarrhea	80 children aged between 3 months and 4 y	Inactivated *Lactobacillus* LB plus fermented culture medium	40% reduction in diarrheal illness duration in the treatment group. Diarrhea duration was significantly reduced in children who had had diarrhea for longer than 24 hours at inclusion (p = 0.044).
Prospective, randomized, double-blind, placebo-controlled trial ^42^	Prevention of diarrhea	377 children aged 12–48 months	Cow’s milk fermented with heat-treated *Lactobacillus paracasei*	The proportion of participants experiencing at least one episode of diarrhea was significantly lower in children receiving cow’s milk fermented with *Lactobacillus paracasei* compared with the placebo (p < 0.0001).
Multicenter, randomized, double-blind, placebo-controlled trial ^43^	Prevention of diarrhea	126 children aged 12–48 months	Cow’s milk fermented with heat-treated *Lactobacillus paracasei*	The proportion of children presenting ≥1 episode of acute gastroenteritis was significantly lower in children receiving *L. paracasei* (p < 0.05) compared with the placebo. They were also 22% less likely than children in the placebo group to experience and episode of diarrhea (p < 0.01).
Exploratory study ^44^	Antibiotic associated diarrhea	184 adults (96 on antibiotics + postbiotic and 88 on antibiotics alone)	Inactivated *Lactobacillus* LB plus fermented culture medium	Less diarrhea in the *Lactobacillus* LB group (antibiotic only: RR = 1.36, 95% CI 1.07–1.72; antibiotic + *Lactobacillus*: RR = 1.16, 95% CI 0.89–1.51, p = 0.046)
Randomized, controlled trial ^48^	Chronic diarrhea	137 adult patients	Inactivated *Lactobacillus* LB plus fermented culture medium	At the 2^nd^ and 4^th^ week, mean bowel frequency was significantly lower in the *Lactobacillus* LB group. At the end of treatment, clinical symptoms were markedly improved in the *Lactobacillus* LB group.
Randomized, double-blind, placebo-controlled trial ^49^	Irritable Bowel Syndrome	443 patients with IBS	*Bifidobacterium bifidum* MIMBb75	*B. bifidum* substantially alleviates IBS and its symptoms compared with the placebo group (risk ratio 1.7, 95% CI 1.3–2.4; p = 0 · 0007).
Functional assessment ^50^	Irritable Bowel Syndrome	297 patients with IBS	Inactivated *Lactobacillus* LB plus fermented culture medium	The average number of stools per week decreased from 17.59 ± 0.6 to 12.83 ± 0.61 after treatment (p < 0.0001). The abdominal pain score on a scale from 1–10 decreased from 4.47 ± 0.15 before treatment to 2.79 ± 0.14 after treatment (p < 0.0001). The bloating score decreased from 4.49 ± 0.18 to 2.56 ± 0.15 (p < 0.0001). The HRQOL score, which is inversely correlated with quality of life, decreased from 5.99 ± 0.14 to 3.92 ± 0.16 (p < 0.0001).
Single center, open-label, prospective, randomized trial^52^	*Helicobacter pylori* infection	120 *H. pylori* positive, dyspeptic volunteers	Heat stabilized *Lactobacillus acidophilus* LB	In the standard treatment group, eradication was successful in 42 of the 58 patients (72%). In the standard treatment plus *L. acidophilus* LB group, the eradication rate increased significantly to 88% (52 of 59 patients) (p = 0.03).

## Overview of clinical benefit evidence organized by mechanism of action

### *Enhancing intestinal barrier function (*Table 1)

#### Evidence of efficacy in gastrointestinal disorders

##### Pediatric population

Acute diarrhea is a significant cause of childhood morbidity and mortality in developing countries^31^ and rotavirus is the most common pathogen causing 29% to 45% of severe diarrhea cases.^32^ Acute gastroenteritis has been found to cause large-scale alterations of the intestinal microbiome.^33^ Enteric bacterial infections markedly reduce the intestinal microbiome richness and diversity which can last up to 14 weeks post-infection.^34^ Similar changes are observed with viral diarrhea.^35^ Microbiome alterations are more significant in children with a “failure to thrive” and these children also take longer to recover from diarrheal illness.^36^ Postbiotics may help to mitigate these alterations and preserve a balanced microbiome during and after diarrheal illnesses. Although live *Lactobacillus* has been shown to be effective against viral diarrhea,^37,38^ studies have also demonstrated that heat-treated *Lactobacillus* LB can promote faster recovery, reduce morbidity and reduce hospitalization duration.^39–41^ Furthermore, well-controlled studies have shown that heat-treated *Lactobacillus paracasei* helps prevent diarrhea by significantly reducing the number of diarrhea episodes compared with a placebo.^42,43^

Antibiotics can also alter the intestinal microbiome leading to antibiotic-associated diarrhea. Although, probiotic efficacy is widely documented with diarrhea and has been suggested to treat antibiotic-associated diarrhea, there is a perception that antibiotics may reduce probiotic viability.^44^ Postbiotics may reduce the risk of antibiotic-associated diarrhea as shown by an exploratory study revealing that in patients receiving antibiotics, there was less diarrhea in the heat-treated *Lactobacillus* LB group.^44^

##### Adult population

Chronic diarrhea is commonly caused by chronic functional diarrhea and chronic parasitic and bacterial infections in developing countries^45^ while in developed countries, irritable bowel syndrome (IBS) is the most common cause affecting up to 15% of adults.^46,47^ Treatment often includes antibiotics and antimotility drugs, but they can be ineffective and cause adverse effects. Postbiotics could be a possible alternative. A recent randomized-controlled study showed that heat-treated *Lactobacillus* LB significantly improved chronic diarrhea and clinical symptoms compared with live lactobacilli (p < 0.05).^48^ Non-viable *Bifidobacterium bifidum* MIMBb75 has been found to substantially alleviate IBS and its symptoms compared with the placebo (p = 0 · 0007).^49^ Similarly, inactivated *Lactobacillus* LB plus fermented culture medium significantly decreased the number of weekly stools (p < 0.0001) and improved abdominal pain, bloating and quality of life in patients with IBS (p < 0.0001).^50^

Postbiotics may also help improve the efficacy of standard treatment for *Helicobacter pylori* infections. Lactobacilli has been shown to inhibit the attachment of *Helicobacter pylori* to gastric epithelial cells in *in vitro* studies.^51^ Furthermore, *L. acidophilus* LB spent culture supernatant decreases *H. pylori* viability, regardless of pH and lactic acid levels, *in vitro* and *in vivo*.^51^ Heat stabilized *L. acidophilus* LB was given to in addition to the standard treatment to *H. pylori* positive patients in an open-label, prospective, randomized trial. It was demonstrated that adding *L. acidophilus* to the standard treatment significantly increases eradication rates compared to standard treatment alone.^52^

**Table 2.** Clinical evidence for postbiotics as microbial therapeutics through immune system modulation.

Study design	Condition	Population	Postbiotic	Results
**Evidence of efficacy in immunity and allergies**				
Randomized, double-blind, placebo-controlled, parallel study ^57^	Examining influence on immune function and quality of life (QoL)	60 healthy adults	Heat-killed *Lactobacillus plantarum* L-137	Compared with a placebo, the change in Con A-induced proliferation and Th1:Th2 ratio were greater in the HK-LP group (p = 0.036 and p = 0.002, respectively). QoL improved more in the HK-LP group than innthe control group (p = 0.049 at week 8, p = 0.092 at week 12)
Randomized, double-blind, placebo-controlled trial ^59^	Perennial allergic rhinitis	90 subjects (adults and children)	Heat-killed *Lactobacillus paracasei* LP33	After 30 d of treatment, quality of life improved more for subjects taking live or heat-killed LP33 in terms of frequency (9.47 ± 2.89, 6.30 ± 2.19, vs. −3.47 ± 1.53, respectively; p < 0.0001) and level of bother (5.91 ± 3.21, 6.04 ± 2.44, vs. −2.80 ± 1.64, respectively; p = 0.004) compared with the placebo group. HK-LP33 efficacy was not inferior to the live variant.
*Ex vivo* cellular study^60^	Grass pollen allergy	Peripheral blood mononuclear cells from 10 adults with grass pollen allergy and 19 non-allergic adults	Inactivated *Lactobacillus LB* and non-pathogenic *Escherichia coli*	CD69 expression on T-lymphocytes was significantly up-regulated by both bacteria (p = 0.001). Allergen stimulation caused significantly increased CD23 expression (p = 0.008) which reduced after Lactobacillus stimulation and significantly reduced with allergen plus E. coli (p = 0.029). Lactobacillus stimulation reduced CD80 expression in the allergic group only (p = 0.021). CD86 expression increased significantly after Lactobacillus stimulation (p = 0.049) and distinctly increased after E. coli in both groups (p = 0.001). Both bacteria modulate allergic immune response through co-stimulatory molecule expression and CD23 alteration. There is clear promotion of T-helper-1 dominated response in allergic participants.
**Evidence of efficacy in upper respiratory tract infections**				
Randomized, single-blind, placebo-controlled study ^63^	Examining the effect of HK L-137 on interferon-β	16 healthy female adults	Heat-killed Lactobacillus plantarum L-137	Interferon-β levels were significantly higher in the HK L-137 group before trivalent influenza vaccination compared with the control group. Vaccination resulted in little additional induction of Interferon-β. Increased type 1 interferon augments host defense against influenza A virus.
Randomized, double-blind, placebo-controlled, parallel study ^65^	Upper respiratory tract infections	78 healthy subjects with high psychological stress levels	Heat-killed Lactobacillus plantarum L-137	Compared with the control group, URTI incidence was significantly lower in the HK L-137 group (p = 0.011). Significant negative correlation between HK L-137 intake duration and URTI incidence, duration, severity, and medication duration. Concanavalin A-induced proliferation of peripheral blood mononuclear cells was significantly greater in the HK L-137 group compared with the control group (p = 0.048).
Prospective, randomized, double-blind, placebo-controlled trial ^42^	Common infectious diseases	377 children aged 12–48 months	Cow’s milk (Group A) or rice (Group B) fermented with *Lactobacillus paracasei* CBA L74	There number of children having at least one common infectious disease was lower in group A (48.2%) and group B (58.5%) than in the placebo group (80.3%). Upper respiratory tract infection incidence was lower in group A (48.2%) and group B (58.5%) compared with the placebo group (70.5%).
Multicenter, randomized, double-blind, placebo-controlled trial ^43^	Common infectious diseases	126 children aged 12–48 months	Cow’s milk fermented with *Lactobacillus paracasei* CBA L74	60% of children in L74 group presented at least one common infectious disease compared with 83% in the placebo group. Numbers of children presenting at least one URTI was significantly lower in the L74 group compared with the placebo (51% vs. 74%, p < 0.05)
Randomized, double-blind, placebo-controlled study ^66^	Viral respiratory tract infections	172 children aged 3 to 6 y	Heat-killed *Pediococcus acidilactici* K15	Salivary IgA levels were maintained significantly higher in the K15 group compared with the placebo. K15 significantly decreased fever duration in children with little intake of fermented food.
Randomized, placebo-controlled, double blind trial ^70^	Influence on levels of salivary secretory IgA	80 elderly participants	Heat-killed *Lactobacillus pentosus* b240	Mean salivary SIgA rate increased steadily until week 4 with a 20% increase compared to week 0. It stayed relatively stable until week 12. The increase in SIgA rate was significantly greater in the b240 group compared with the placebo group.
Randomized, double-blind, placebo-controlled trial ^71^	Common cold	280 elderly adults	Heat-killed *Lactobacillus pentosus* b240	The common cold incidence rate was 47.3% for the placebo group, 34.8% for the low-dose group and 29.0% for the high dose group (P for trend = 0.012). Quality of life increased dose-dependently.

### *Immune system modulation (*Table 2)

#### Evidence of efficacy in immunity and allergies

Live *Lactobacillus* spp. have been shown to improve immune function and health-related quality of life (HRQoL), and control allergies. This may be due to its ability to skew the immune system away from T helper 2 (Th2) responses towards T helper 1 (Th1) responses.^53^ This may be beneficial in Westernized societies where good public hygiene and fewer infections reduce this response, increasing the risk of developing allergies.^54^ Poor immunity also increases the risk of pathogenic infections and can reduce HRQoL.^55,56^

There is also well-controlled clinical evidence demonstrating that heat-treated *Lactobacilli plantarum* (HK-LP) may also improve Th-1 related immune function. Compared with a placebo, the change in Con A-induced proliferation and Th1:Th2 ratio was greater (p = 0.036 and p = 0.002, respectively) and HRQoL improved more in the HK-LP group (p = 0.049 at week 8, p = 0.092 at week 12).^58^

Similarly, live *Lactobacillus paracasei* 33 (LP33) has been shown to effectively and safely improve QoL for patients with house-dust mite induced perennial allergic rhinitis.^59^ A subsequent randomized-controlled trial revealed that heat-treated LP33 had similar efficacy to the live variant since patients taking either the live or heat-treated LP33 had improved QoL scores for frequency (p < 0.0001) and level of bother (p = 0.004) compared with the placebo.^60^ Furthermore, inactivated *Lactobacillus* LB and non-pathogenic *E. coli* have been shown to modulate allergic immune response in grass pollen allergies, both clearly through Th1-dominated responses.^61^

Allergic rhinitis requires long-term management so the efficacy, safety, low cost, and ease of storage may mean that postbiotics are a good treatment option. It has also been suggested that postbiotics could potentially improve food allergies.^62^

#### Evidence of efficacy in upper respiratory tract infections

Animal and clinical data support the use of three postbiotic strains (*Lactobacillus plantarum, Pediococcus acidilactici*, and *Lactobacillus pentosus)* in preventing upper respiratory tract infections (URTI).

In mice, heat-treated *Lactobacillus plantarum* L-137 has been shown to stimulate type 1 interferon production thus enhancing protection against the influenza virus^63^ which has also been observed in humans.^64^ It has been suggested that high levels of psychological stress increase the risk of acute respiratory illness.^65^ Well-controlled clinical evidence suggests daily heat-treated *Lactobacillus plantarum* L-137 (HK L-137) intake can decrease URTI incidence in healthy people with high levels of psychological stress, possibly through immune function augmentation. The 12-week randomized-controlled study found URTI incidence was significantly lower in the HK L-137 group vs the control group (p = 0.011). There was also a significant negative correlation between HK L-137 intake duration and URTI incidence, duration, severity, and medication duration. The concanavalin A-induced proliferation of peripheral blood mononuclear cells was significantly greater in the HK L-137 group than in the control group (p = 0.048).^66^ This means HK L-137 intake could potentially reduce this URTI risk.

Similarly, studies have evaluated the possibility of preventing respiratory tract infections in children. *Lactobacillus paracasei* CBA L74 was found to reduce the number of cases of pharyngitis, laryngitis and tracheitis in two well-controlled trials including children aged 12–48 months.^42,43^ Furthermore, *Pediococcus acidilactici* K15 was found to support anti-infectious immune systems in children who ate less fermented foods and maintained salivary secretory IgA levels in all subjects in a randomized-controlled study focusing on respiratory tract infection prevention in pre-school children. The four-month study also found that in children eating little fermented food, K15 significantly reduced fever duration compared with the placebo.^67^

Salivary IgA levels decrease with age^68–70^ suggesting that elderly adults may be more susceptible to upper respiratory tract infections. *Lactobacillus pentosus* b240 has been shown to increase salivary IgA levels^71^ and a well-controlled trial demonstrated that b240 intake significantly reduces common cold incidence rates in elderly adults, possibly improving infection resistance through mucosal immunity. This study found that common cold incidence rates were lower (log-rank test, p = 0.034) and general health perceptions, determined using SF-36 w, dose-dependently increased (p for trend = 0.016).^72^

**Table 3.** Clinical evidence for postbiotics as microbial therapeutics through multiple mechanisms of action.

Study design	Condition	Population	Postbiotic	Results
**Evidence for efficacy in stress and neurological conditions**				
Randomized, double-blind, placebo-controlled study ^82^	Stress	60 medical students preparing for national examinations	Heat-inactivated *Lactobacillus gasseri* CP2305	Anxiety and sleep disturbance were significantly reduced in the CP2305 group compared with the placebo group. CP2305 also significantly shortened sleep latency and wake time after sleep onset. Salivary chromogranin A levels were also significantly lowered in the CP2305 group compared with placebo. CP2305 was also found to attenuate stress-induced decline of *Bifidobacterium* spp. and elevation of *Streptococcus* spp.
Multicenter, randomized, double-blind, placebo-controlled trial ^84^	Mild Alzheimer’s Disease	328 patients aged 60 to 85 y with mild Alzheimer’s Disease	Plasmalogens	The Wechsler Memory Scale-Revised improved significantly in the treatment group. In a subgroup analysis, this scale also improved significantly in females and participants under 77 y of age. The between group difference for females was statistically significant (p = 0.017), as it was for those under 77 y of age (p = 0.029). Plasma plasmalogen decreased significantly more in the placebo group compared with the treatment group.
**Evidence for efficacy in metabolic syndrome**				
Randomized, double-blind, placebo-controlled pilot study ^99^	Obesity-related disorders	32 overweight/obese adults with insulin resistance	Pasteurized *Akkermansia muciniphila*	Insulinemia (−34.08 ± 7.12%, P = 0.006), plasma total cholesterol (−8.68 ± 2.38%, P = 0.02) and body weight (−2.27 ± 0.92 kg, P = 0.091) were reduced and insulin sensitivity improved (+28.62 ± 7.02%, P = 0.002) in the pasteurized *A. muciniphila* group compared with placebo. Compared with the baseline, fat mass (−1.37 ± 0.82 kg, P = 0.092) and hip circumference (−2.63 ± 1.14 cm, P = 0.091) decreased in the pasteurized *A. muciniphila* group.

### *Multiple mechanisms of action (*Table 3)

#### Evidence for efficacy in stress and neurological conditions

The gut-brain axis has been shown to have a crucial role in maintaining intestinal homeostasis and brain function.^73,74^ The intestinal microbiome affects communication between the intestines and the brain through immune, endocrine and neural pathways,^75^ and evidence suggests that it significantly impacts brain function affecting mood, recognition and behavior.^76^

Probiotics have been shown to modulate hippocampus-mediated negative feedback regulation of the hypothalamic–pituitary–adrenal axis, mitigate stress-induced pain and behavior,^77,78^ transduce signals to the brain via the afferent vagal nerve and relieve mood disturbances.^79,80^ Research is underway to investigate the effects of heat-treated bacteria.

Well-controlled clinical results show that long-term *Lactobacillus gasseri* CP2305 use may improve the mental state, sleep quality and gut microbiome of healthy adults under stressful conditions. The 24-week randomized-controlled study included students preparing for national medical examinations and daily CP2305 significantly reduced anxiety, sleep disturbance and salivary chromogranin A levels compared with the placebo (p < 0.05). CP2305 also attenuated the stress-induced decline of *Bifidobacterium* spp. and the stress-induced elevation of *Streptococcus* spp.^81^

Postbiotics have uses in other nervous system-related conditions. For example, it has been demonstrated in mice that daily intake of plasmalogens, a component of postbiotics, can inhibit memory loss by inhibiting glial activation.^82^ Furthermore, a well-controlled study revealed that plasmalogen may improve cognitive function in patients with mild Alzheimer’s disease.^83^

Potential uses include new anti-depressant approaches since depression has been linked with intestinal microbiome alterations.^84,85^ Furthermore, multiple sclerosis pathogenesis has also been linked with microbiome alterations^86^ including lower levels of SCFA-producing bacteria^87^ which could be a potential target for postbiotics.

#### Efficacy in cardiac and vascular disorders

Oxidative damage can lead to inflammation and cardiovascular disease. Molecules with antioxidant properties found in postbiotic preparations could therefore potentially reduce this.^88^ Inflammation and endothelial dysfunction occurring with chronic kidney disease (CKD) may cause cardiac damage; however, postbiotic preparations have been identified as a potential prophylaxis for CKD-associated cardiac damage.^89^ Further data suggest that bacterial metabolites and components may prevent or slow the progression of CKD and hypertension.^89^

#### Evidence for efficacy in metabolic syndrome

Metabolic syndrome is characterized by various comorbidities predisposing individuals to cardiovascular pathologies and type 2 diabetes mellitus.^90^ Obesity-related disorder onset can be linked to the intestinal microbiome.^91^ Modulating the intestinal microbiome with postbiotics has been shown to facilitate weight reduction^92^ and may reduce cholesterol, as demonstrated in one study where *B. longum* administered to rats had a cholesterol-lowering effect.^93^ Moreover, muramyl dipeptide, a bacterial wall component, can modulate GLP-1 secretion thereby increasing insulin sensitivity and improving glucose tolerance.^94^

In rodents, live *Akkermansia muciniphila* reduced obesity, glucose intolerance, insulin resistance, steatosis, and gut permeability.^95–97^ Subsequently, it was discovered that pasteurization enhances its effect on adiposity, insulin resistance and glucose tolerance.^96^ Clinical data from a randomized-controlled study including overweight/obese, insulin-resistant individuals show that pasteurized *A. muciniphila* reduces liver dysfunction and inflammation blood marker levels while leaving the overall gut microbiome structure unaffected. The three-month study found that pasteurized *A. muciniphila* was safe and well tolerated, improved insulin sensitivity (p = 0.002), and reduced insulinemia (p = 0.006), plasma total cholesterol (p = 0.02) and body weight (p = 0.091) compared with the placebo. It also reduced fat mass (p = 0.092) and hip circumference (p = 0.091) compared with baseline.^98^

Similarly, the intestinal microbiome has been shown to also influence appetite,^99^ meaning postbiotics could potentially be used as an appetite regulator.

## Future potential

There are areas where postbiotics have potential efficacy, for example the liver. The gut–liver axis is a bidirectional relationship between the gut, its microbiome, and the liver. The portal vein carries gut-derived products to the liver and the liver feedbacks via bile and antibody secretion into the intestine. A healthy microbiome maintains gut-liver axis homeostasis. Liver cirrhosis is associated with a profoundly altered microbiome and damaged intestinal barrier. There is increasing evidence that microorganism-derived metabolites including trimethylamine, SCFAs and ethanol have a pathogenic role in non-alcoholic fatty liver disease. Postbiotics are considered a potential new therapeutic avenue for these liver diseases, but more research is needed to confirm their benefit.^100^

Probiotic genome editing already exists^101^ and could potentially be used with postbiotics to modify the precursor bacteria, thus generating new postbiotic interventions. Furthermore, advancements in microbiome metagenomic mapping can further elucidate interactions between commensal microorganisms and the intestine as well as strengthen the evidence for postbiotics. This could even be extended to individualized microbiome phenotyping to prevent disease, though this is a long way off.

Establishing the interaction mechanisms between postbiotics and commensal microorganisms will improve the understanding of the potential clinical benefits and possibly lead to targeted postbiotic therapy.

## Conclusion

Postbiotics are safe and stable with a long shelf-life enabling easy storage and transportation and can be administered during antibiotic treatment without affecting efficacy, making them an appealing alternative to probiotics. There is growing evidence for the clinical benefits of postbiotics in the management of highly prevalent conditions including gastrointestinal, dermatological, and neurological disorders as well as respiratory infections and metabolic syndrome. Postbiotics may offer a novel therapeutic approach for these conditions and could be a safer alternative to probiotics, particularly in vulnerable populations such as pediatrics. Additional randomized, placebo-controlled clinical trials are necessary to further verify the clinical benefits of postbiotics.

## Data Availability

Data sharing is not applicable to this article as no new data were created or analyzed in this study.
